# Unmasking Parasitic Colitis Mimicking Inflammatory Bowel Disease

**DOI:** 10.7759/cureus.102310

**Published:** 2026-01-26

**Authors:** Ethan Shamsian, Kranthi K Mandava, Joshua E Pagán-Busigó, Michael Bebawy, Ahmed Al-Khazraji

**Affiliations:** 1 Internal Medicine, Rutgers University New Jersey Medical School, Newark, USA; 2 Gastroenterology and Hepatology, Rutgers University New Jersey Medical School, Newark, USA

**Keywords:** autoimmune hepatitis, cirrhosis, eosinophilic colitis, inflammatory bowel disease, parasitic infection, schistosomiasis, strongyloidiasis

## Abstract

Parasitic infections may clinically mimic inflammatory bowel disease (IBD), particularly in patients with underlying liver cirrhosis, creating substantial diagnostic uncertainty. We describe a 27-year-old Egyptian male with presumed autoimmune hepatitis (AIH)-related cirrhosis who presented with abdominal pain and bloody diarrhea. Endoscopic evaluation suggested IBD; however, histopathology demonstrated eosinophilic colitis. Subsequent stool and serologic testing confirmed co-infection with *Schistosoma* *mansoni* and *Strongyloides stercoralis*. Treatment with antiparasitic therapy resulted in marked clinical improvement. This rare case of dual parasitic infection underscores the importance of detailed migration and residence history in endemic regions and freshwater exposure history, as these parasites can cause presinusoidal portal hypertension and progressive hepatic fibrosis. Parasitic etiologies should remain in the differential diagnosis for gastrointestinal symptoms in patients from endemic regions to avoid misdiagnosis and potentially harmful immunosuppressive therapy. Overall, these findings suggest that the parasitic infection likely contributed significantly to the patient’s hepatic fibrosis, potentially mimicking or exacerbating the presumed AIH-related cirrhosis rather than representing purely isolated autoimmune disease.

## Introduction

Patients with liver cirrhosis frequently develop cirrhosis-associated immune dysfunction, a state of immune dysregulation that increases susceptibility to bacterial, viral, and parasitic infections [1]. Parasitic infections such as schistosomiasis and strongyloidiasis may clinically resemble inflammatory bowel disease (IBD), often creating significant diagnostic and therapeutic challenges [2-7]. Additionally, schistosomiasis itself can cause hypergammaglobulinemia and autoantibody positivity, which may confound diagnostic testing and contribute to the misdiagnosis of autoimmune hepatitis (AIH) in cirrhotic patients. Importantly, cirrhosis-associated immune dysfunction and portal hypertension may also blunt peripheral eosinophilic responses through splenic sequestration, immune exhaustion, and altered cytokine signaling, further obscuring the diagnosis of parasitic disease.

Hepatic schistosomiasis is characterized by granulomatous inflammation surrounding schistosome eggs within the portal venous system, leading to elevated presinusoidal portal pressure and portal hypertension in the absence of established cirrhosis. Persistent inflammation may ultimately progress to periportal fibrosis and the development of true cirrhosis. In patients with pre-existing liver disease, superimposed parasitic infection can further exacerbate hepatic dysfunction and precipitate clinical decompensation [8].

We report a case of poly-parasitic infection in a male patient with presumed AIH-related cirrhosis whose clinical presentation mimicked IBD and was complicated by progressive cirrhotic decompensation.

## Case presentation

A 27-year-old male originally from Egypt with a history of liver cirrhosis presumed secondary to AIH presented with several days of persistent abdominal pain and bloody diarrhea. He reported being diagnosed with AIH in 2016 after testing positive for antinuclear antibody (ANA) and anti-smooth muscle antibody (ASMA) with an immunoglobulin G (IgG) level >3,000 mg/dL; specific ANA and ASMA titers were not available for review. A liver biopsy performed in 2016 demonstrated grade 4 severe widespread interface hepatitis with grade II focal hepatocellular necrosis and stage I portal fibrosis, supporting the diagnosis of AIH at that time; no schistosome ova or pipestem fibrosis were identified. He was subsequently diagnosed with cirrhosis in 2022 in Egypt after developing ascites, hemorrhoids, abdominal pain, and gastrointestinal bleeding.

On admission, laboratory evaluation demonstrated elevated liver enzymes, hypoalbuminemia, and iron-deficiency microcytic anemia (Table 1). Peripheral eosinophil count was within normal limits.

**Table 1:** Admission laboratory findings in a patient with parasitic colitis and cirrhosis

Laboratory Test	Patient Value	Reference Range
Hemoglobin (g/dL)	9.9	13.5-17.5
Mean Corpuscular Volume (fL)	68.5	80-100
Serum Iron (µg/dL)	12	60-170
Ferritin (ng/mL)	12	30-400
Alanine Aminotransferase (U/L)	61	7-56
Aspartate Aminotransferase (U/L)	80	10-40
Alkaline Phosphatase (U/L)	376	44-147
Albumin (g/dL)	2.4	3.5-5.0
Peripheral Eosinophils	Normal	0-500 cells/µL
Immunoglobulin G (mg/dL)	>3000	700-1600

**Figure 1:**
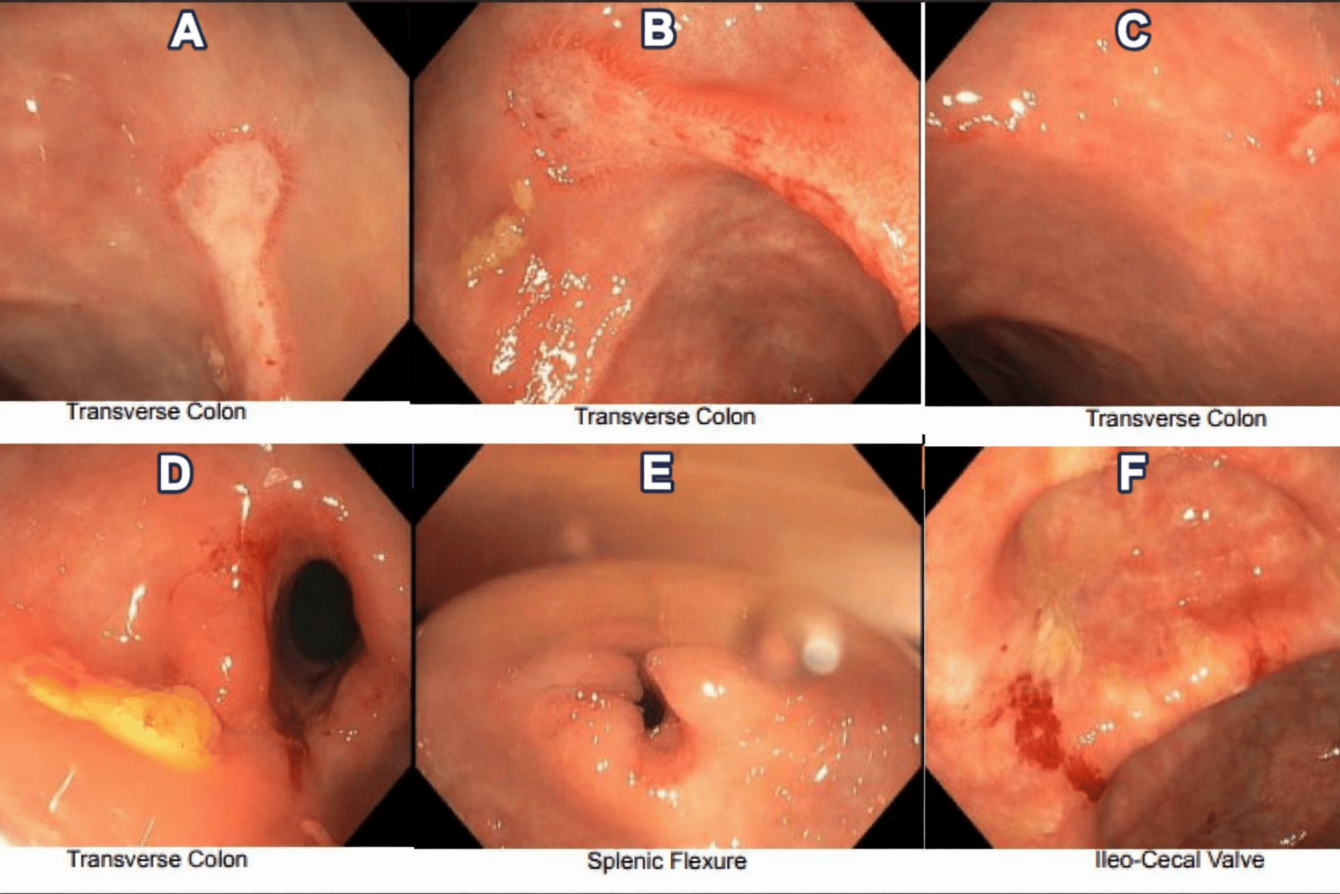
Colonoscopy findings in a 27-year-old cirrhotic male with parasitic colitis mimicking IBD Colonoscopy images showing large ulcerations within strictures in the mid-to-distal transverse colon (A-D), a stricture at the splenic flexure (E), and a friable ileocecal valve (F). IBD, inflammatory bowel disease

Esophagogastroduodenoscopy (EGD) demonstrated grade I esophageal varices without stigmata of active bleeding. Colonoscopy revealed moderate strictures in the mid and distal transverse colon, multiple ulcerations within the mid-transverse colon, including within a stricture, and friable mucosa with contact bleeding extending from the transverse colon to the cecum (Figure 1).

Given the presence of skip lesions, colonic strictures, and a reported history of oral ulceration, IBD was initially considered. Targeted biopsies were obtained, and serum and stool studies were performed to evaluate alternative infectious and inflammatory etiologies, including a viral hepatitis panel, Epstein-Barr virus serologies, cytomegalovirus testing, and stool PCR for common enteric pathogens. All of these studies were negative, making common bacterial and viral causes of colitis less likely and prompting further evaluation for parasitic infection. Histopathology demonstrated chronic active eosinophilic colitis with crypt architectural distortion and marked eosinophilic infiltration of up to 80 eosinophils per high-power field; however, no ova or parasites were visualized in the colonic biopsy specimens. Inflammatory markers, QuantiFERON-Gold testing, and human leukocyte antigen B27 (HLA-B27) were all negative, lowering suspicion for IBD and spondyloarthropathy-associated colitis.

Given the patient’s childhood exposure to freshwater lakes in schistosomiasis-endemic regions of Egypt, additional parasitic evaluation was pursued, including stool ova and parasite examination and *Strongyloides* serology, which confirmed infection with *Schistosoma mansoni* and *Strongyloides stercoralis*. He was treated with praziquantel and albendazole, with adjunctive corticosteroids administered after initiation of antiparasitic therapy to control severe eosinophilic colitis and inflammatory symptoms, resulting in significant clinical improvement. He was scheduled for repeat EGD with endoscopic ultrasound (EUS) and colonoscopy with biopsy to monitor treatment response.

## Discussion

This case describes a 27-year-old Egyptian male with presumed AIH-related cirrhosis who presented with abdominal pain and bloody diarrhea. Colonoscopy revealed friable mucosa with moderate stricturing and segmental ulceration, initially raising concern for IBD. However, histopathology demonstrated marked eosinophilic infiltration, and stool and serologic testing confirmed dual infection with *S. stercoralis* and *S. mansoni*. These findings prompted reconsideration of the underlying etiology of his chronic liver disease. Although AIH was initially believed to be the primary cause of cirrhosis, the identification of schistosomiasis, together with the known association of chronic schistosomiasis with hypergammaglobulinemia and autoantibody positivity, raises the possibility that his liver disease was at least partially driven by schistosomiasis and may have been misclassified as autoimmune. While the 2016 liver biopsy demonstrated interface hepatitis consistent with AIH, the later recognition of endemic exposure and dual parasitic infection supports reappraisal of the original diagnostic attribution and suggests that schistosomiasis may have contributed significantly to portal hypertension and hepatic fibrogenesis.

This dual parasitic infection is exceedingly rare and, to our knowledge, represents the first reported case in which co-infection with *S. stercoralis* and *S. mansoni* produced significant colonic strictures and ulcerations mimicking IBD in the setting of portal hypertension and cirrhosis. The overlapping gastrointestinal manifestations created a substantial diagnostic challenge, particularly in the presence of advanced liver disease. Notably, the absence of peripheral eosinophilia may have delayed diagnosis, as eosinophilic responses are often blunted in patients with cirrhosis or immunosuppression [9].

Parasitic infections are well-recognized mimickers of chronic gastrointestinal disorders. Several features may help distinguish parasitic colitis from idiopathic IBD. Parasitic infections often demonstrate patchy or segmental involvement with relative rectal sparing, marked eosinophilic infiltration on histology, and submucosal granulomas centered on ova or larvae, whereas IBD more typically shows crypt architectural distortion, basal plasmacytosis, and continuous mucosal inflammation in ulcerative colitis or transmural lymphoid aggregates in Crohn’s disease [10]. Strongyloidiasis has been mistaken for IBD or carcinoid syndrome, amebiasis for colorectal malignancy or Crohn’s disease, and schistosomiasis for IBD or cryptogenic cirrhosis [2-7]. These diagnostic pitfalls are especially relevant in cirrhotic patients, whose susceptibility to infection is heightened by cirrhosis-associated immune dysfunction, including impaired reticuloendothelial clearance, decreased hepatic protein synthesis, and dysfunction of gut-associated lymphoid tissue [11,12].

Among parasitic pathogens, *Schistosoma* species play a particularly well-established role in hepatic fibrogenesis. In hepatosplenic schistosomiasis, chronic immune activation in response to egg deposition promotes granuloma formation and periportal fibrosis. Even before established cirrhosis, egg-mediated inflammation increases presinusoidal resistance, leading to early portal hypertension. Continued egg deposition stimulates a Th2-dominant immune response with production of interleukin (IL)-33 and IL-4, activation of M2 macrophages and hepatic stellate cells, and downstream profibrotic signaling through mediators such as IL-13, transforming growth factor beta-1 (TGF-β1), and vascular endothelial growth factor (VEGF) [8]. This cascade results in progressive collagen deposition and architectural distortion, eventually culminating in cirrhosis and increased risk of hepatocellular carcinoma. Additional profibrotic mechanisms, including microRNA dysregulation and damage-associated molecular patterns, may further amplify liver injury [8]. In this patient, schistosomiasis likely accelerated fibrosis in conjunction with AIH, contributing to early portal hypertension and cirrhosis. 

Simultaneous infection with multiple parasites is exceptionally uncommon. Bhattacharyya et al. reported a 50-year-old Yemeni man with chronic gastrointestinal and respiratory symptoms who was ultimately found to have *Entamoeba histolytica*, *S. mansoni*, *Fasciola hepatica*, and *S. stercoralis* infections [13]. That diagnosis required repeated testing in the setting of eosinophilia and hepatosplenomegaly. In contrast, our patient lacked dermatologic or pulmonary manifestations of strongyloidiasis, suggesting that cirrhosis-associated immune dysfunction may have facilitated parasitic persistence or reactivation. His childhood exposure to freshwater sources in an endemic region and prior diarrheal illness further support the possibility of latent infection reactivating with immune compromise. 

Importantly, in patients with suspected or confirmed *S. stercoralis* infection, corticosteroid therapy poses a unique and well-documented risk of precipitating hyperinfection syndrome, a potentially fatal complication characterized by uncontrolled autoinfection, disseminated larvae, and secondary gram-negative sepsis [14]. For this reason, antiparasitic therapy with ivermectin or albendazole must be initiated before or concomitantly with corticosteroids when immunosuppression is clinically necessary. In the present case, corticosteroids were administered only after antiparasitic coverage was established, specifically to manage severe eosinophilic colitis and inflammatory symptoms, thereby mitigating the risk of *Strongyloides* hyperinfection while allowing symptomatic control.

Finally, this case mirrors observations from endemic regions where patients presenting with IBD-like symptoms were ultimately diagnosed with parasitic colitis on histopathologic evaluation [15,16]. These reports emphasize the importance of a detailed travel and environmental exposure history and highlight that negative stool studies do not exclude invasive parasitic disease. In patients from endemic areas, parasitic infection should remain a critical consideration before initiating immunosuppressive therapy.

## Conclusions

This case underscores the critical importance of considering parasitic infections in the differential diagnosis of IBD-like presentations, particularly in patients with chronic liver disease originating from endemic regions. Dual infection with *S. mansoni* and *S. stercoralis* can produce severe colonic inflammation, stricturing disease, and progressive hepatic fibrosis, closely mimicking autoimmune and inflammatory etiologies. Failure to recognize these infections may lead to inappropriate initiation of immunosuppressive therapy without adequate antiparasitic coverage, with potentially life-threatening consequences.

In the era of global migration, we recommend that patients from schistosomiasis- or strongyloidiasis-endemic regions who are labeled with AIH, cryptogenic cirrhosis, or IBD-like colitis undergo targeted parasitic screening. This is particularly critical prior to initiating or escalating immunosuppressive therapy. A careful exposure history combined with early parasitic testing should be incorporated into diagnostic algorithms in non-endemic, high-resource settings to avoid diagnostic delay, guide appropriate therapy, and prevent irreversible liver injury or *Strongyloides* hyperinfection syndrome.
